# The Analysis of Oral and Fecal Virome Detects Multiple Novel Emerging Viruses in Snakes

**DOI:** 10.1155/2023/4214812

**Published:** 2023-05-17

**Authors:** Aijing Liu, Zhige Tian, Chuanming Yin, Jie Zou, Shan Wu, Yi Luo, Xin Chen, Yi Dai, Siyi Yang, Yanxi Li, Tongyu Li, Peng Guo, Xiaoliang Hu

**Affiliations:** ^1^Faculty of Agriculture, Forestry, and Food Engineering, Yibin University, Yibin Animal and Plant Inspection and Quarantine Engineering Technology Research Center, Yibin Key Laboratory of Zoological Diversity and Ecological Conservation, Yibin, China; ^2^Department of Preventive Veterinary Medicine, College of Veterinary Medicine, Qingdao Agricultural University, Qingdao, China; ^3^Clinical Medicine Department, Harbin Medical University, Orthopaedics Department, Fourth Affiliated Hospital of Harbin Medical University, Harbin, China; ^4^Liuzhou Integrated Chinese and Western Medicine Snake Injury Treatment Center, Liuzhou Traditional Chinese Medicine Hospital, Liuzhou, China

## Abstract

Wild animals are considered reservoirs for emerging and reemerging viruses, such as the novel severe acute respiratory syndrome coronavirus 2 (SARS-CoV-2). Previous studies have reported that bats and ticks harbored variable important pathogenic viruses, some of which could cause potential diseases in humans and livestock, while viruses carried by reptiles were rarely reported. Our study first conducted snakes' virome analysis to establish effective surveillance of potential transboundary emerging diseases. Consequently, *Adenoviridae*, *Circoviridae*, *Retroviridae*, and *Parvoviridae* were identified in oral samples from *Protobothrops mucrosquamatus*, *Elaphe dione*, and *Gloydius angusticeps* based on sequence similarity to existing viruses. *Picornaviridae* and *Adenoviridae* were also identified in fecal samples of *Protobothrops mucrosquamatus*. Notably, the iflavirus and foamy virus were first reported in *Protobothrops mucrosquamatus*, enriching the transboundary viral diversity in snakes. Furthermore, phylogenetic analysis revealed that both the novel-identified viruses showed low genetic similarity with previously reported viruses. This study provided a basis for our understanding of microbiome diversity and the surveillance and prevention of emerging and unknown viruses in snakes.

## 1. Introduction

Reptiles are highly diverse, including more than 1,200 genera and 11,000 species [1], which were classified into four orders: Squamata (containing 10,417 species of lizards, snakes, and amphisbaenians), Testudines (containing 351 turtle and tortoise species), Crocodylia (containing 24 crocodiles, alligator, caiman, and gavial), and Rhynchocephalia species [2]. Currently, 205 snake species are found in mainland China [3]. Snakes are natural carriers of a large variety of viruses, including ranavirus (*Chondropython viridis*) [4], erythrocytic necrosis virus (*Thamnophis sauritus*) [5], herpesvirus (*Boa constrictor*) [6], adenovirus (*Python regius*) [7, 8], parvovirus (*Pantherophis guttatus*) [9], circovirus (*Aspidites melanocephalus*) [10], retrovirus (*Vipera russelli*) [11], reovirus (*Python regius*) [12], paramyxovirus (*Bothrops alternatus*) [13–15], calicivirus (*Crotalus unicolor*) [16], Japanese encephalitis virus (*Zaocys dhumnades*) [17], western equine encephalitis virus (*Thamnophis* spp.) [18], picornavirus (*Zamenis lineatus*) [19], eastern equine encephalitis virus (*Agkistrodon piscivoru*) [20], and chikungunya virus (*Python regius*) [21]. Besides, snakes could be a potential intermediate reservoir for SARS-CoV-2, based on the relative synonymous codon usage bias resembling snakes compared with other animals [22, 23]. Some of the pathogens above could be transboundary viruses (e.g., JEV and EEEV), which could also infect humans or other animals, seriously threatening public health and ecological balance.

Next-generation sequencing (NGS) technology (e.g., pyrosequencing and sequencing-by-synthesis) [24] has been widely applied in virology, including the metagenomic characterization of viruses in ticks, wild rodent feces, bat feces, and human nasopharyngeal aspirates and feces [25–28]. NGS could detect and identify multiple pathogens simultaneously and be applied in diagnosing mixed infections and detecting unknown pathogens [29]. Besides, as the emergence of atypical symptoms of various diseases has been increasing in recent years, NGS could be an efficient tool for updating the diagnostic strategy in pathogen diagnosis [30].

This study collected fecal and oral samples of three snake species (*Protobothrops mucrosquamatus*, *Elaphe dione*, and *Gloydius angusticeps*) from Sichuan Province, China, for metagenomic analysis. The viromes of these three species were characterized based on sequence-independent polymerase chain reaction (PCR) amplification and NGS. Results revealed the complete or partial genome sequences of five novel viruses with low similarity to known viruses. These genomic data extended our knowledge of viral diversity and evolution in snakes. Further studies are needed to understand these viruses' pathogenicity and clinical impact. Our study provides the foundation for further research on the virome in cold-blooded animals and contributes to maintaining the ecological balance in ravine and forest habitats in the mountains. A preprint of this study has previously been published [31].

## 2. Materials and Methods

### 2.1. Ethics Approval

Our experimental procedures complied with the current laws of China for the care and use of experimental animals and were approved by the Animal Research Ethics Committee of Yibin University. No animals were sacrificed specifically for this study.

### 2.2. Sample Collection

**Figure 1:**
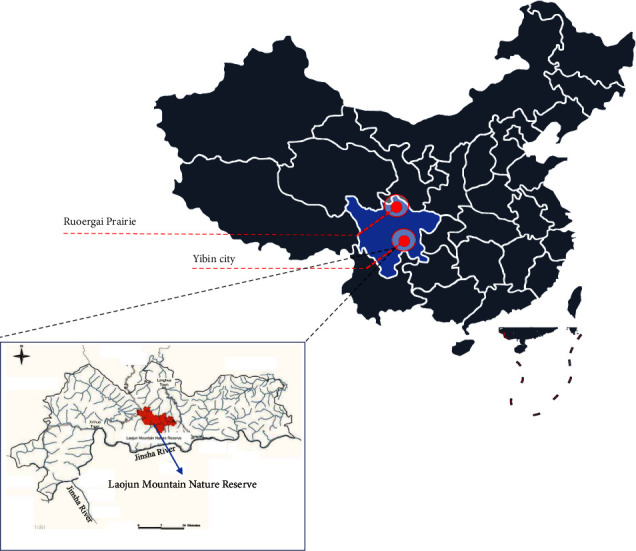
Map of Laojun Mountain Nature Reserve.

From July to September 2020, four brown-spotted pit vipers (*Protobothrops mucrosquamatus*) and three dione rat snakes (*Elaphe dione*) were captured from ravine and forest habitats in the Laojun Mountains, 110 km from Yibin city (average altitude: 300–600 m) of Sichuan provence. Three *Gloydius angusticeps* were captured from grassland and lakeside habitats in the Ruoergai Prairie (average altitude: 3,300–3,600 m) in Sichuan (Figure 1). The 10 snakes were individually placed in sterilized tubs overnight to collect pharyngeal and anal swab samples. Animals were individually placed in sterilized tubs, and their skins were cleaned with 75% alcohol to prevent sample contamination. All samples were collected opportunistically with sterilized swabs in areas where snakes were captured. The swabs were placed in RNase-free tubes and immediately transported on dry ice to Shanghai Biozeron Biotechnology Co., Ltd. (China) the same day. The snakes were then released back into the wild.

### 2.3. RNA and DNA Extraction

Total RNA was extracted from tissue using TRIzol® (Invitrogen) following the manufacturer's instructions. The complementary DNA was synthesized using random hexamer primers, M-MLV Reverse Transcriptase. According to the manufacturer's protocols, DNA was extracted from 10 fecal and oral samples using an EZNA® Stool DNA Kit (Omega BioTek, Norcross, GA, USA). The DNA libraries were constructed and sheared with a fragment length of 450 bp using a Covaris S220 Focused-Ultrasonicator (Woburn, MA, USA). Raw sequencing reads were processed to improve the validity of reads for further analysis using Trimmomatic (https://www.usadellab.org/cms/uploads/supplementary/Trimmomatic) [32]. Reads were then mapped against the human genome (version: hg19) using the BWA-MEM algorithm (parameters: -M -k 32 -t 16, https://bio-bwa.sourceforge.net/bwa.shtml).

### 2.4. Library Preparation and Illumina HiSeq Sequencing

The transcriptome was analyzed and described as previously described [33]. Briefly, to select cDNA fragments of 200–300 bp in length, the libraries were purified and amplified with universal PCR primers and index primers by Phusion DNA polymerase (NEB) using PCR assay. The library quality was assessed, and the library preparations were sequenced on Illumina HiSeq 4000 platform by NovoGene (Beijing). Paired-end reads were generated with 150 bp. Raw paired-end reads were verified using Trimmomatic parameters (SLIDINGWINDOW: 4:15 MINLEN: 75) (v0.36 https://www.usadellab.org/cms/uploads/supplementary/Trimmomatic). 200.0 Gb of paired-end reads were obtained for all the samples. Clean data from all samples were used for assembly with MEGAHIT (https://www.l3-bioinfo.com/products/megahit.html). Metagenome binning of contigs from each sample was performed using metaBAT2 [34]. Completeness and contamination of bins were determined using CheckM v1.0.3 [35]. MetaSPAdes was used for 4. Metagenomic assembled genomes (MAGs) re-assembly with clean reads using the BWA-MEM method to improve the assembly quality of MAGs [36]. All genes in all bins were transformed and used for phylogenetic tree reconstruction using PhyloPhlAn [37].

### 2.5. Detection of Specific Viruses in Fecal and Oral Samples from Snakes

Specific primers were designed based on iflavirus, adenovirus, circovirus, foam virus, and parvovirus sequences. The primers are listed in Table 1. Before sequencing, positive amplification products were cloned into the pMD18-T vector (TaKaRa). Three independent clones were sequenced and verified.

### 2.6. Complete Genome Sequencing of Iflavirus

Extraction of RNA and generation of cDNA were performed as previous described [38]. In total, 12 pairs of primers were developed for PCR amplification, as described in Table 1.

### 2.7. Sequence Alignment and Phylogenetic Analysis

Sequence data were assembled and analyzed using ClustalX and DNASTAR. Phylogenetic trees based on whole-genome sequences were constructed in MEGA (v6.0) with the maximum-likelihood (ML) method. Bootstrap values were estimated for 1,000 replicates.

### 2.8. Nucleotide Sequence Accession Numbers

The complete sequence PMP20 obtained in this study has been submitted to the GenBank database (accession number: MZ005704). The accession numbers for PM-LJS-1 and PM-LJS-5, ED-LJS-4, PM-LJS-3, ED-LJS-6, GA-LJS-8 are OP644553 to OP644558, respectively.

## 3. Results

### 3.1. Solexa Sequencing and General Virome of Snakes

After removing the barcode and host gene sequences, 3.42 × 10^8^ reads were obtained by Solexa sequencing with an average length of 823 nucleotides (nt).  Table 2 shows 3,218,295 and 1.48 × 10^8^ reads were annotated to eukaryotes and bacteria, respectively, while 225,014 reads were matched to viruses, including Retroviridae, *Adenoviridae*, *Caliciviridae*, *Circoviridae*, and *Parvoviridae*. A total of 2,038 assembled contigs (>100 nt) were constructed and compared with GenBank. Among them, 967 contigs were homologous to phages, and 1,071 were homologous to eukaryotic viruses. The longest contig was 4,769 nt. Most configs showed low similarities with the protein sequences of known viruses, suggesting that these sequences represent novel viruses.

### 3.2. Detection and Identification of Iflavirus

Iflaviruses belong to the family *Iflaviridae* (order *Picornavirales*) and contain positive-stranded RNA genomes between 9 and 11 kb in length [39]. Iflavirus genome organization is monopartite and monocistronic, encoding capsid proteins at the 5′ end and replicase proteins at the 3′ end [40]. All classified iflaviruses species infect arthropod hosts, mostly insects, including honeybees [41], planthoppers [42], soybean thrips [43], mites [44], and mosquitoes [45, 46].

Here, we identified an iflavirus from the feces of *Protobothrops mucrosquamatus*, denoted as YB-PMP20. The YB-PMP20 genome was 9,808 nt in length, with an A, G, T, and C nucleotide composition of 2,827, 2,329, 2,680, and 1,972, respectively. The G + C content in the YB-PMP20 genome was 43.85%, higher than that reported for other iflaviruses, including Vespa velutina-associated ifla-like virus (VVAILV) (35.71%), Aedes Ifla-like virus (AEIV) (36.42%), Culex picorna-like virus (CUPV) 1 (36.75%), Fitzroy Crossing iflavirus (FCIV) 1 (37.83%), Darwin bee virus (DABV) 2 (36.39%), and Sanxia water strider virus (SWSV) 8 (37.06%), but lower than that of Lygus lineolaris virus (Lylv) 1 (46.15%) (Table 3). The genome contained a 5′-untranslated region (UTR), followed by a single open reading frame spanning 8,988 nt from position 304 to 9,291, and a 3′-UTR. A consensus invertebrate initiation sequence (ANNAUGG; N = any nucleotide) was located at nucleotide position 301–307 nt, and a translation initiation codon (AUG) was located at nucleotide position 304–306 nt. The 2,995-amino acid (aa) polyprotein had a calculated molecular mass of 335.3 kDa, an isoelectric point of 6.709, and a charge of −8.611 at pH 7.0. Alignment of the polyprotein sequences of YB-PMP20 and iflavirus showed the highest sequence identity with Hubei picorna-like virus (HUPV) 36 (99.2% in nt and 99.6% in aa), LyIV-1 (46.6% in nt and 20.9% in aa), VVAILV (58.6% in nt and 48.6% in aa), and AEIV (43.7% in nt and 24.6% in aa) (Table 3).

**Figure 2:**
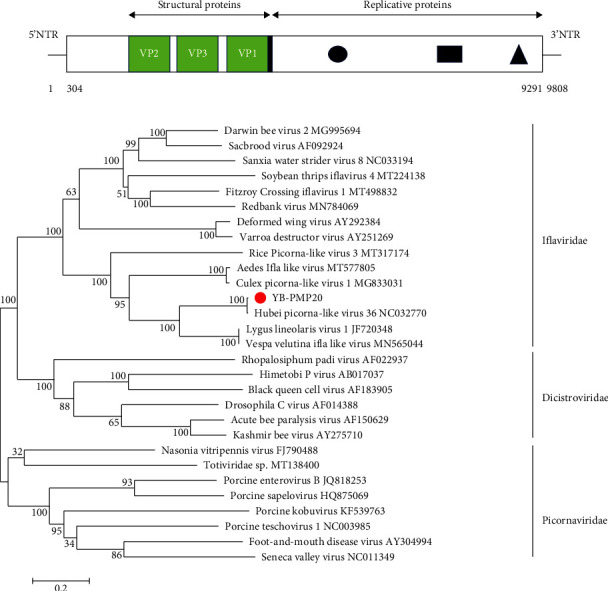
(a) Schematic of YB-PMP20 genome (not drawn to scale). (●) represents helicase, (■) represents protease, and (▲) represents RdRp. (b) Maximum-likelihood phylogenetic tree based on genomic sequences of iflavirus.

The peptide domains of the YB-PMP20 capsid protein, helicase, peptidase, and RNA-directed RNA polymerase (RdRp) were identified through similarity searches using the Simple Modular Architecture Research Tool (SMART: https://smart.embl-heidelberg.de), Conserved Domain Database (CDD: https://www.ncbi.nlm.nih.gov/Structure/cdd/cdd/shtml), and SWISS-MODEL Bioinformatics server [47, 48]. The YB-PMP20 polyprotein amino acid regions 130–382, 429–680, and 747–992 were identified as capsid proteins VP2, 3, and 1, respectively, which shared low protein sequence identity (30%) with the sacbrood virus. The conserved VP4-VP3 cleavage site (N_X_/D_X_P) has been confirmed in Iflavirus [49]. However, the deduced VP4 was not found in the consensus sequences (Figure 2).

**Figure 3:**
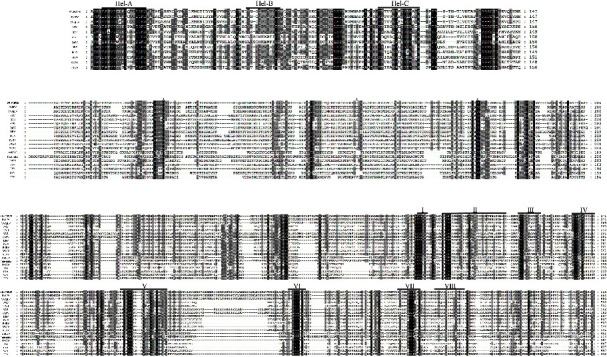
Alignment of conserved amino acid motifs of helicase (a), protease (b), and RdRp (c).

RNA helicase domains were identified in the polyprotein from 1,437–1,583 aa and showed 25.93% amino acid identity with 2C helicase from enterovirus 71 (EV71) and 2C ATPase from picornavirus (Figure 3). Three conserved helicase motifs (A, B, and C) are present in picornaviruses and iflaviruses [50]. The highly conserved amino acids within motif A (GxxGxxGKS) and motif B (QxxxxxDD) were identified in the YB-PMP20 sequence between aa 1,449–1,456 and 1,495–1,503. In YB-PMP20, the amino acids within motif C were KKxxxxPxxxxxATN, in contrast to the consensus motif KGxxxxSxxxxxSTN [51]. Protease was identified at aa 2,174–2,379 and showed 22.29% amino acid identity with the 3C protease from coxsackievirus [52]. The putative residues H^2217^, E^2290^, and C^2340^ may form the catalytic triad in the protease of PMP20. RdRp domains were identified at aa 2,416–2,974 and showed 20.86% amino acid identity with the RdRp of Sapporo virus. Eight conserved RdRp amino acid motifs are found in RNA viruses [50]. The putative RdRp conserved domains of PMP20 are shown in Figure 3(c). A highly conserved domain (^2526^TSxGxP^2631^) was found in PMP20 prior to motif I of the RdRp domain [53]. Using the ML approach, we constructed a phylogenetic tree based on the full sequences of iflaviruses. The iflaviruses clustered into a large clade consisting of two subclades. The phylogenetic tree indicated that YB-PMP20 was clustered in the same subclade as HUPV 36, LyIV-1, and VVAILV (Figure 2). These findings may contribute to understanding the evolution of iflaviruses in snakes.

### 3.3. Detection and Identification of Adenovirus

**Figure 4:**
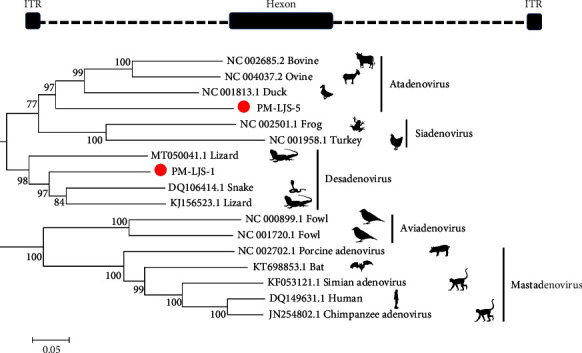
Phylogenetic analysis of hexon of adenovirus. The sequences in our study are identified by a red circle.

Previous research indicated that members of the family Adenoviridae could infect a variety of vertebrates, including mammals, birds, fish, and amphibians, and cause various diseases [54, 55]. Adenoviruses can also cause respiratory infection and subclinical to lethal symptoms in reptiles [56]. In this study, two adenoviruses were identified in oral and fecal samples of *Protobothrops mucrosquamatus* by PCR. The hexon genes were amplified, and two sequences were confirmed, i.e., PM-LJS-5 (1 254 bp) and PM-LJS-1 (2 736 bp), respectively. Sequence and phylogenetic analyses based on the hexon genes showed that PM-LJS-5 and PM-LJS-1 were divergent from current adenoviruses, with 70% identity with snake and lizard adenoviruses. PM-LJS-1 was closely clustered with lizard and snake adenoviruses, forming a unique clade. In the oral samples, PM-LJS-5 was closely related to but differed from adenovirus, indicating they were different isolates (Figure 4).

### 3.4. Detection and Identification of Circovirus

**Figure 5:**
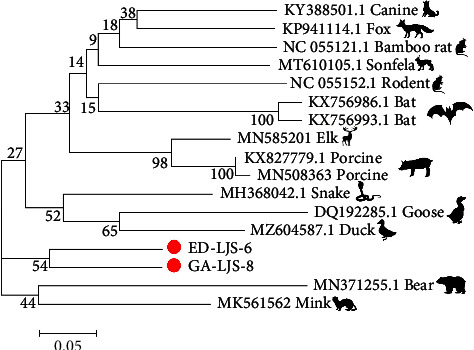
Phylogenetic analysis of complete sequences of circovirus. The sequences in our study are identified by a red circle.

Circoviruses have been found in human fecal samples, wild chimpanzees, bats, and snakes [10, 57–59]. Based on Solexa sequencing, we identified two sequences in oral samples from *Elaphe dione* (ED-LJS-6), and *Gloydius angusticeps* (GA-LJS-8), which were 164 nt and 605 nt in length, respectively, and contained in Rep. Phylogenetic analysis showed that the two circovirus sequences shared 67% nt identity with all known circoviruses, indicating they were different isolates (Figure 5). The two sequences formed a unique cluster and shared 60% and 67% identity with the only known snake circovirus, indicating that they were novel isolates.

### 3.5. Detection and Identification of the Foamy Virus

**Figure 6:**
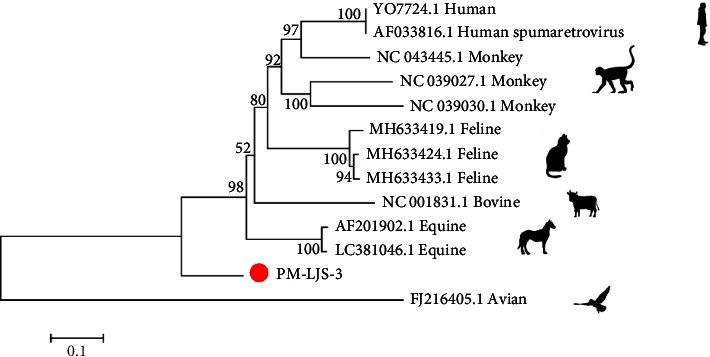
Phylogenetic analysis of complete sequences of foamy virus. The sequences in our study are identified by red circle.

Foamy viruses belong to the *Retroviridae* family and can infect cattle, cats, horses, gorillas, monkeys, and humans [60]. This study identified and designated a foamy virus sequence in Protobothrops mucrosquamatus as PM-LJS-3. A partial genomic sequence (859 nt) that encoded the N terminus of the *pol* gene was also detected in this study. The partial *pol* gene of PM-LJS-3 showed 70% nucleotide acid identity with the *pol* genes of other foamy viruses. We constructed a phylogenetic tree based on the complete sequences of PM-LJS-3 and other foamy viruses (Figure 6). The results indicated that PM-LJS-3 had low genomic similarity with other foamy viruses that infect other hosts.

### 3.6. Detection and Identification of Parvovirus

**Figure 7:**
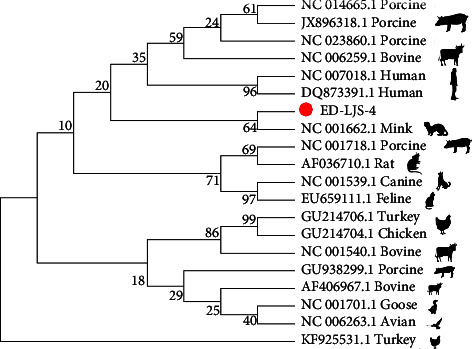
Phylogenetic analysis of complete sequences of parvovirus. The sequences in our study are identified by red circle.

Parvovirus has been detected in reptiles, named Dependovirus, which was found in the intestinal epithelium [9, 56]. In our study, we identified one sequence (ED-LJS-6) from the oral samples of *Elaphe dione* using PCR, which showed a nucleotide acid identity of 76% to NS1 of parvovirus (Figure 7). The phylogenetic tree based on complete parvovirus sequences indicated that ED-LJS-6 (106 nt) was clustered with mink parvovirus.

## 4. Discussion

Snakes are widely distributed in the world and are common predators of monkeys [61], shell snails [62], kangaroos [63], fish [64], leeches [64], earthworms [64], frogs [64], tadpoles [64], fish eggs [65], lizards [66, 67], field voles [66], and shrews [66]. Along with more than 15 different viruses, various zoonotic diseases have also been confirmed in snakes [22, 68]. Previous studies also showed that snakes might act as reservoirs for transboundary viruses [69, 70]. Three snakes were captured from the southwest of Sichuan Province for metagenomic analysis. All snake habitats were located within or close to the nature reserve to facilitate identifying and tracking viral communities in wildlife. In this study, we first detailed the viromes of three kinds of snakes in China from oral and fecal samples and identified various viruses in *Adenoviridae*, *Circoviridae*, *Retroviridae*, *Parvoviridae*, and *Picornaviridae*, providing a basis for our general understanding of microbiome diversity.

According to the NGS data taxonomic assignment results, we directly conducted the alignments with the valid reads. The assembled contigs were then used in subsequent genome sequencing to facilitate reads-based PCR. Various viruses in bats, ticks, and humans have been identified using metagenomic analyses [25, 28, 58, 71–74]. In the present study, we first identified and characterized an iflavirus strain (YB-PMP20) from the brown-spotted pit viper, which showed characteristics typical of the *Iflaviridae* family, including capsid protein, helicase, protease, and RdRp domains. Sequence analysis indicated that YB-PMP20 was similar to HUPV 36, LyIV-1, and VVAIV in *Diptera* spp., *Lygus lineolaris*, and *Vespa velutina nigrithorax*, respectively, raising the question of how this insect virus exists in snakes. We speculated that (1) snakes may occasionally prey on insects, resulting in transmission of the virus from insects to snakes, and (2) amphibians, including frogs, may feed on insects, resulting in viral transmission from insects to amphibians to snakes. Thus, these results indicate that snakes could be a reservoir of insect and amphibian pathogens.

Previous studies showed that adenoviruses were found in the harbor in bats with wide geographic distribution [72]. Adenoviruses can cause lethargy, neurological disorder, esophagitis, and gastroenteritis in snakes [19]. Here, we identified two adenoviruses, PM-LJS-1 and PM-LJS-5, in the fecal and oral samples of *Protobothrops mucrosquamatus*, respectively. PM-LJS-1 was closely related to adenoviruses from lizards and snakes, while PM-LJS-5 was clustered with the adenoviruses. These results suggest that multiple adenoviruses are prevalent in snakes. The adenoviruses of bats and snakes identified in China have different ancestors.

This study is the first to identify a foamy virus (PM-LJS-3) in snakes, suggesting that members of the *Retroviridae* family can infect reptiles and mammalian hosts. Previous studies have shown that foamy viruses can exhibit cross-species transmission, with potential risk to humans [75, 76]. The foamy virus was also identified in Chinese bats. However, no consistent sequence alignment results in analyzing the evolution and origin of the foamy virus in China. Thus, further research is required to monitor widely distributed snake species closely.

The previous study isolated parvovirus and circovirus strains in corn snakes (*Elaphe guttata*) and black-headed pythons [9, 10]. Here, we identified parvovirus and circovirus sequences in *Elaphe dione* (ED-LJS-4 and ED-LJS-6, respectively) and a circovirus sequence in *Gloydius angusticeps* (GA-LJS-8). Phylogenetic analysis showed that ED-LJS-4 was closely related to mink parvovirus, and the two circovirus sequences formed a unique cluster among other circoviruses. Interestingly, *Gloydius angusticeps* live in high-altitude localities with limited human exposure. These results suggest that greater attention should be paid to snakes and snake-borne viruses to monitor potential disease outbreaks in other wildlife.

This study had three main limitations. First, we did not determine the sex and age of the snakes, which might affect the hunting behavior. Second, the concentration of nucleic acid in feces and oral cavities was low, and the sequences were not completely amplified and assembled. Third, the identified viruses were not successfully isolated. Thus, more factors, including sex, age, behavior, season, and location, should be considered, and virus isolation and whole-genome sequencing should also be carried out in the experiment.

In conclusion, we explored the viromes of three snake species in China. Two viruses (adenovirus and iflavirus) were identified in fecal samples from *Protobothrops mucrosquamatus*. In oral samples from *Protobothrops mucrosquamatus*, *Elaphe dione*, and *Gloydius angusticeps*, four viruses (adenovirus, circovirus, foamy virus, and parvovirus) were detected. Notably, this study identified the iflavirus and foamy virus in snakes (*Protobothrops mucrosquamatus*) for the first time, and most identified viruses were distinct from any known virus, including bat-borne viruses. These results suggest that snakes may serve as reservoirs for multiple viral diseases. Thus, more attention should be paid to the relationship between snakes and snake-associated viruses, and further studies are required to characterize viruses and pathogenesis in snakes.

## Figures and Tables

**Table 1:** Primers for identifying and sequencing the targeted sequences.

	Name	Sequence (5′-3′)	Position
Iflavirus	1F	TTCTTACCCCAAAGGTAGGA	1–20
	1R	CAACCAGCTTAAATTCTACA	990–1009
	2F	CTTTTCATTGAGGATCCCAG	900–919
	2R	CTTAACAATGTCAAACTCAT	1970–1989
	3F	TGATATTGCTAAGAAGCAGA	1800–1819
	3R	ATTTTTTGGTCGAATAAGTT	2870–2889
	4F	CAGCACCAAATAACTAAGAT	2746–2765
	4R	ACTTCACCCAGATCTGTCAA	3727–3746
	5F	GCATACGATCTATGATCTTA	3471–3490
	5R	ATTCCTAACGAACAAGAAAG	4268–4287
	6F	TTGGCAAAATACTTAAGCAG	4034–4053
	6R	TTAACTAAGACGTCTCGTCT	5014–5033
	7F	CTGAGCTCTACCAGCTGAAG	4856–4875
	7R	AACAAGCTCATAATTACAAC	5842–5861
	8F	TGTAGCAAAATCTCCTGTGG	5677–5696
	8R	ATATCGCTGTAGTTTTCAGC	6514–6533
	9F	GCCTTGTAACACTACTGATT	6387–6406
	9R	GCTCTAATTCTAGCTCCAGT	7405–7424
	10F	TAGTGCATATGATGAACTGA	7242–7261
	10R	AGCGAAGGTGAGAAGTAAAA	8205–8224
	11F	GGAGGCAAGGAATTTTAGCA	8054–8073
	11R	CATATGATAGCAATAGGCTA	9081–9100
	12F	TCTCCAAAACATGTCATTTT	8922–8941
	12R	TTTATATTGTTTTTGTATTT	9789–9808
Adenovirus	ADE-F1	ATGGAGCCGCAGCGTGAGTT	
	ADE-R1	TCATGTCGCCGCCGAGCCCG	
	ADE-F2	TGGACAATGAAAACCCGTTC	
	ADE-R2	TTCTAGTGGGTTGATTGATA	
Parvovirus	PA-F	ACTCCAACAATGAAAACT TT	
	PA-R	ATAGCTTTAAATTCTTTAAC	
Foamy virus	FO-F	TATTTTTAAGGACTTTGATT	
	FO-R	ATAAAAAGGTCTGACTAGTT	

Circovirus	CIR-F1	AACTGTGTCTATGCAATTGT	
	CIR-R1	ACAAACTCCATAGTGCCTCC	
	CIR-F2	ATACTTGATGATTTTTATCG	
	CIR-R2	CAATCTTCACTATACCATTC	

**Table 2:** Overview of Solexa sequencing.

Pool	Datesize (nt)	Reads	Contigs	A. length	Eukaryotic	Bacterial	Virus-like
PDY	28611987	51491382	37769	2703	234324	19384699	1064
LJS-1	67315710	27714252	761127	497.415	650151	4718859	77203
LJS-2	63880587	39182981	629161	464.155	486001	18276106	63946
LJS-3	71080937	28793902	722020	516.55	146057	11660467	9592
LJS-4	83663608	26110389	101522	580.145	173409	15834919	9663
LJS-5	88838540	27797398	50633	560.58	137339	16159340	4693
LJS-6	76047551	39507966	102727	535.655	171782	28801253	11734
LJS-7	90501243	17737928	75617	643.07	201026	4438947	13408
LJS-8	101601196	37060828	172874	548.51	408994	11789563	13092
LJS-9	96756160	15200070	130527	577.545	222964	4297182	7650
LJS-10	86284497	31362454	136134	616.48	386248	12204637	12969
Total	854582016	341959550	2920111	8243.105	3218295	147565972	225014

**Table 3:** Comparison of amino acid and nucleotide sequence identity between YB-PMP20 and another representative iflaviruses.

	YB-PMP20		VVIV		AEIV		CUPV1		DABV2		FCIV1		HUPV36		LyIV1		REBV		SABV		SWSV		STIV4	
	nt%	aa%	nt%	aa%	nt%	aa%	nt%	aa%	nt%	aa%	nt%	aa%	nt%	aa%	nt%	aa%	nt%	aa%	nt%	aa%	nt%	aa%	nt%	aa%
Polyprotein	100	100	58.6	48.6	43.7	24.6	44.1	24.5	37.1	22.7	36.8	22.3	99.2	99.6	46.6	20.9	36.8	22.9	37.1	22.7	36.1	22.2	37	23.3
VP1	100	100	62.6	56.4	42	27	42.4	26.2	29.1	31.2	32.9	28.4	99.2	98.8	47.5	29.7	30.5	27.9	30.0	27.9	30.8	28.9	30.6	28.1
VP2	100	100	64.3	65.2	40.1	25.7	41.4	25.7	35.4	20	31.6	17.4	99.5	99.6	49.3	21.2	34.3	21.9	34.5	18.7	32.8	23.2	36.4	25
VP3	100	100	64.1	64.3	43.1	28.5	43.3	28.1	36.3	26.8	38.6	29.6	99.3	100	46.1	29	36.4	26.8	35.2	27.6	33.7	29.8	38.4	27.7
Helicase	100	100	70.2	78.9	34.4	29.3	36.3	29.3	45.8	40.1	50.2	37.4	99.1	100	53.9	42.1	49.8	38.8	46.7	40.8	47.5	36.3	47.4	40.1
Protease	100	100	58.6	50	47.4	25.6	46.9	25.6	40.6	22.8	38.1	22.9	99.5	100	50.5	27	37.6	22.9	37.0	22.8	36.7	23.8	35.8	22.4
RdRp	100	100	58.6	56.5	47.4	40.1	46.9	40.5	40.6	36.1	38.1	32.9	99.5	99.8	45.3	33.2	37.6	34.3	37.0	37	36.7	33.6	35.8	34.9

## Data Availability

The complete sequence PMP20 obtained in this study has been submitted to the GenBank database (accession number: MZ005704). The accession numbers for PM-LJS-1, PM-LJS-5, ED-LJS-4, PM-LJS-3, ED-LJS-6, and GA-LJS-8 are OP644553 to OP644558, respectively.
